# Supplementary material for: Kinesin-6 regulates cell-size-dependent spindle elongation velocity to keep mitosis duration constant in fission yeast
Source: eLife. 2019 Feb 26;8:e42182. doi: 10.7554/eLife.42182 (PMC6391065; doi:10.7554/eLife.42182)
Supplement: Figure 1—source data 1. — Mean values (bold) and corresponding standard deviations of cell length, total spindle duration and spindle length, duration and velocity for each mitotic phase: phase I (prophase), phase II (metaphase-anaphase A) and phase III (anaphase B) of wee1-50 (n = 53), wild-type (n = 61) and cdc25-22 cells (n = 60). Data obtained from n analyzed cells was collected from three independent experiments. [file elife-42182-fig1-data1.docx]

| **Cell type** | **Cell length**  **(µm)** | **Spindle length (µm)** | | | **Duration (min)** | | | **Velocity (µm/min)** | | | **Total spindle duration (min)** |
| --- | --- | --- | --- | --- | --- | --- | --- | --- | --- | --- | --- |
|  |  | I | II | III | I | II | III | I | II | III |  |
| ***wee1-50*** | **10.55**  ±1.01 | **2.41**  ±0.46 | **2.51**  ±0.54 | **10.14**  ±1.21 | **9.10**  ±2.49 | **6.76**  ±4.13 | **14.07**  ±2.49 | **0.21**  ±0.06 | **0.04**  ±0.09 | **0.59**  ±0.12 | **30**  ±4.19 |
| **Wt** | **13.32**  ±0.66 | **2.29**  ±0.44 | **2.74**  ±0.52 | **11.93**  ±0.89 | **7.48**  ±2.03 | **7.13**  ±2.18 | **14.26**  ±1.34 | **0.25**  ±0.07 | **0.09**  ±0.06 | **0.70**  ±0.07 | **28.74**  ±2.10 |
| ***cdc25-22*** | **24.66**  ±2.62 | **2.84**  ±0.67 | **3.66**  ±0.79 | **17.34**  ±1.37 | **8.13**  ±2.41 | **5.15**  ±2.42 | **16.8**  ±2.67 | **0.29**  ±0.06 | **0.16**  ±0.14 | **0.89**  ±0.1 | **30.33**  ±2.5 |
