# Supplementary material for: Kinesin-6 regulates cell-size-dependent spindle elongation velocity to keep mitosis duration constant in fission yeast
Source: eLife. 2019 Feb 26;8:e42182. doi: 10.7554/eLife.42182 (PMC6391065; doi:10.7554/eLife.42182)
Supplement: Figure 4—source data 1. — Mean values and corresponding standard deviations of total Klp9-GFP intensity, Klp9-GFP intensity in anaphase spindles and the ratio of total intensity and nuclear volume, referred to as Klp9 concentration. Data obtained from n analyzed cells (wee1-50: n = 48, wt: n = 46, cdc25-22: n = 30) was collected from three independent experiments. [file elife-42182-fig4-data1.docx]

| **Cell type** | **total Klp9-GFP intensity (AU)** | **Klp9-GFP intensity (midzone) (AU)** | **c (Klp9)** |
| --- | --- | --- | --- |
| ***wee1-50*** | **989** ± 212 | **433** ± 94 | **5.95** ± 1.81 |
| **wt** | **1619** ± 428 | **533** ± 103 | **5.07** ± 1.63 |
| ***cdc25-22*** | **2515** ± 613 | **1155** ± 323 | **5.55** ± 1.35 |
