# Supplementary material for: Kinesin-6 regulates cell-size-dependent spindle elongation velocity to keep mitosis duration constant in fission yeast
Source: eLife. 2019 Feb 26;8:e42182. doi: 10.7554/eLife.42182 (PMC6391065; doi:10.7554/eLife.42182)
Supplement: Figure 5—source data 1. — Mean values and corresponding standard deviations of anaphase B spindle elongation velocity and GFP-klp9 intensity at the midzone of anaphase spindles in wee1-50, wild-type and cdc25-22 cells expressing klp9 under the control of nmt promoters with different strength. Data was collected from three independent experiments. [file elife-42182-fig5-data1.docx]

| **Cell type** | ***klp9* expression** | **V (anaphase B)**  **(µm/min)** | **Klp9-GFP intensity (midzone) (AU)** |
| --- | --- | --- | --- |
| ***wee1-50*** | **control** | **0.59 ± 0.12** | **49 ± 25** |
|  | ***pnmt81-klp9*** | **0.71 ± 0.12** | **163 ± 70** |
|  | ***pnmt41-klp9*** | **0.78 ± 0.11** | **275 ± 111** |
|  | ***pnmt1-klp9*** | **1.06 ± 0.15** | **1636 ± 554** |
| **wt** | **control** | **0.7 ± 0.08** | **83 ± 26** |
|  | ***pnmt81-klp9*** | **0.81 ± 0.09** | **152 ± 65** |
|  | ***pnmt41-klp9*** | **0.9 ± 0.1** | **436 ± 186** |
|  | ***pnmt1-klp9*** | **1.06 ± 0.14** | **1931 ± 800** |
| ***cdc25-22*** | **control** | **0.92 ± 0.1** | **113 ± 34** |
|  | ***pnmt81-klp9*** | **0.93 ± 0.09** | **514 ± 242** |
|  | ***pnmt41-klp9*** | **1.05 ± 0.1** | **780 ± 293** |
|  | ***pnmt1-klp9*** | **1.16 ± 0.1** | **6984 ± 3375** |
