# Supplementary material for: Kinesin-6 regulates cell-size-dependent spindle elongation velocity to keep mitosis duration constant in fission yeast
Source: eLife. 2019 Feb 26;8:e42182. doi: 10.7554/eLife.42182 (PMC6391065; doi:10.7554/eLife.42182)
Supplement: Figure 6—source data 1. — Mean values and corresponding standard deviations of Ase1-GFP intensity and Ase1-GFP signal length in wee1-50, wild-type and cdc25-22 cells. Data obtained from n analyzed cells (wee1-50: n = 24, wt: n = 28, cdc25-22: n = 30) was collected from three independent experiments. [file elife-42182-fig6-data1.docx]

| **Cell type** | **Ase1-GFP**  **Intensity (midzone) (AU)** | **Ase1-GFP**  **signal length (µm)** |
| --- | --- | --- |
| ***wee1-50*** | **575 ± 147** | **3.74 ± 0.73** |
| **wt** | **756 ± 179** | **3.62 ± 0.87** |
| ***cdc25-22*** | **1437 ± 383** | **4.45 ± 0.73** |
